# Enhancing Safety in Reconstructive Microsurgery Using Intraoperative Indocyanine Green Angiography

**DOI:** 10.3389/fsurg.2019.00039

**Published:** 2019-07-02

**Authors:** Ingo Ludolph, Raymund E. Horch, Andreas Arkudas, Marweh Schmitz

**Affiliations:** Department of Plastic and Hand Surgery and Laboratory for Tissue Engineering and Regenerative Medicine, University Hospital of Erlangen, Friedrich-Alexander-University of Erlangen-Nuernberg FAU, Erlangen, Germany

**Keywords:** ICG, Indocyanine Green angiography, microsurgery, free flap, imaging

## Abstract

Intraoperative assessing and postoperative monitoring of the viability of free flaps is of high relevance in reconstructive microsurgery. Today different methods for the evaluation of tissue perfusion are known. Indocyanine Green angiography is an emerging technique among plastic surgeons with a broad scope of applications especially in microsurgical free flap transfer. We demonstrate the value and clinical application of this technique based on representative selected cases where Indocyanine Green angiography was used in microsurgical free flap transfers from different anatomic donor sites during the operation. Hereby perforator selection, flap tailoring, changes of blood flow and patency of anastomoses was judged and decision making was based on the angiographic findings. This method has proven to be valid, reproducible and easy to use. The application is not limited to the evaluation of skin perfusion, but is also applicable to muscle tissue or chimeric or composite flaps. Reliable judgement is especially given for the extent of arterially perfused tissue following complete flap dissection. Moreover, this real-time angiography revealed a high sensitivity for the detection of poorly perfused flap areas, thus supporting the conventional clinical judgement and reducing complications. In summary Indocyanine Green angiography has the potential to reduce flap related complications and to contribute to enhancing and extending the possibilities of free flap surgery.

## Introduction

In plastic reconstructive surgery, the number of free tissue transfers has increased significantly in recent decades. Given the appropriate indication recent advancements in operative techniques and imaging modalities have facilitated microvascular reconstructions to become safer, more reliable procedures almost independent from the patient's age (1–4).

Preoperative assessment of the microvasculature anatomy at the tissue harvesting site with advanced imaging modalities has assisted surgeons in the appropriate selection of the donor site, perforator, and flap and has led to an overall improvement in the flap outcomes (1, 5). Furthermore, the question of the intraoperative decision making especially in critically large free flaps, the individual intraoperative perforator constellation and the patency of anastomoses is as relevant as the question of the postoperative flap monitoring. Since the early days of microsurgical reconstructive procedures postoperative flap monitoring in particular is of high relevance for a successful outcome. Hence many clinical and experimental studies deal with this issue. Therefore, modern imaging devices ideally should address the entire scope of a reconstructive procedure, from the planning to the postoperative perfusion monitoring. Today several different techniques are available, all of them with the aim of decreasing flap associated complications.

Laser assisted Indocyanine Green angiography (ICG angiography) in its advanced technical version provides real-time angiography, which enables decision making on tissue perfusion in free and pedicled flaps to be made with high reliability both intraoperatively and postoperatively. ICG was introduced into clinical routine in the 1950's. It was initially used in hepatology as a function test, in cardiologic diagnostics and later by ophthalmologists as it shows less leakage from blood vessels as compared to fluorescein (6, 7). In ophthalmology it is valuable because it remains for a long time in more blood-perfused tissues such as the choroid and the blood vessels.

In this study we give insight into the spectrum of the application of ICG angiography in plastic reconstructive surgery based on representative and selected clinical cases and demonstrate the advantages and disadvantages of this procedure in the context of the current literature.

## Materials and Methods

Among the variety of more than 150 free flaps and pedicled flaps performed in our institution per year four representative clinical cases with different indications for a reconstructive procedure at varying anatomical locations were selected to highlight the status of ICG angiography.

Written informed consent was obtained from all patients in accordance with the Declaration of Helsinki and ICG measurements were performed within a study protocol which was approved by the institutional Review Board (registration number 85_13 B).

As a method for skin perfusion analysis we used ICG-angiography (SPY Elite System, Novadaq Technologies Inc., Toronto, Canada) which has been utilized by our group in more than 300 cases since 2014. Exclusion criteria were solely based on contraindications for the application of Indocyanine Green, e.g., iodine allergy, autonomous adenoma of thyroid gland, hyperthyroidism or due to refusal by the patient. In all free flaps, independent of the anatomic region or the tissue included, it was routinely performed in a standard mode using a 12.5 mg bolus of ICG dye through a venous line.

After intravenous injection of ICG dye, it binds to plasma proteins. The short plasma half-life period of ICG of 3 to 4 min allows repeated injections. It is not metabolized and is excreted by the liver into bile. At the area of interest the ICG is excited by a near-infrared laser. The laser device has no potential for causing damage to the tissue or the observer. The fluorescent substance displays an absorption maximum at 805 nm and an emission maximum at 835 nm. Light at a wavelength of 800 nm in the near-infrared range is minimally absorbed by water or hemoglobin and is not scattered by tissues, which allows visualization of blood vessels (7). The dye highlights vessels up to a minimum of 3 to 5 mm in the tissue. The perfusion pattern, the intensity of the fluorescence as an indicator for dye uptake and the clearance within the tissue is displayed on a video monitor. Well-perfused areas appear bright due to dye uptake, whereas mal-perfused areas are relatively dark. In a standard mode the device displays the video in a gray scale. An additional analysis tool provides color modes and calculation of ingress and egress rates in absolute or relative values. Furthermore, contour levels can be defined for determination of perfusion areas in relation to an automatically or individually set references point in the field of interest. The analysis tool allows the user to work on the videos and pictures at a later time.

## Results

### Case 1

**Figure 1:**
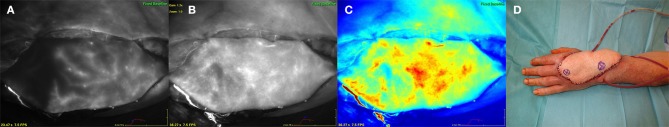
Free anterior lateral thigh flap (ALT) for reconstruction of the dorsum of the left hand. **(A)** (left) ALT flap after harvesting at the left thigh. ICG angiography marking the perforasome perfused by the proximal perforator revealing a distinct perfusion border. **(B)** (middle left) Flap perfused via a proximal and distal perforator showing perfusion of adjacent perforasomes. **(C)** (middle right) ICG angiography color mode. **(D)** (right) ALT flap after inset at the left hand. Asterix within the flap marking the distal (right) and proximal (left) perforator.

A 71 year old male patient suffered from an extensive tissue defect at the dorsum of the left hand as a result of a bicycle incident. After multiple debridement and wound conditioning using negative pressure wound therapy the defect had to be reconstructed by a large free anterior lateral thigh flap from the left thigh. During flap harvesting two distant main perforators were detected, located very lateral within the flap. Following complete flap dissection the first ICG measurement was performed with the flap left in place at the thigh. Thus, the special perforator constellation and the borders of the adjacent perforasomes could be determined. Figure 1 clearly displays the perforasome border nourished by the proximal perforator in the flap which was not to judge sufficiently by clinical signs. After a few seconds the distal perforasome was also perfused shown by an uptake of the ICG dye. Due to this analysis both perforators were then included in the flap.

The flap was anastomosed to the left radial artery in an end-to-side fashion as well as to concomitant recipient veins. Hereupon another ICG measurement revealed a well-perfused flap without changes of blood flow pattern compared to the point after flap harvesting. The perforasome constellation was confirmed and the flap exactly trimmed to the defect size dependent on the ICG perfusion pattern (Figure 1).

### Case 2

**Figure 2:**
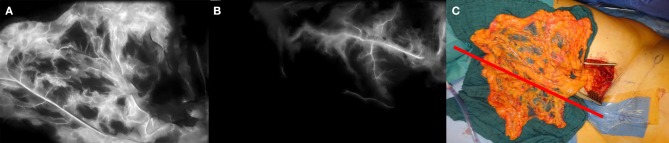
Free omentum majus flap at the right inguinal region. **(A)** (left) ICG angiography of the omentum majus after anastomosis to the femoral vessels showing exact perfusion of the vessel arcades and the adjacent tissue. **(B)** (middle) Mal-perfused part of the omentum majus flap. **(C)** (right) Flap at the inguinal region after anastomosis. Red bar indicates the border between well-perfused (top) and mal-perfused (bottom) parts of the omentum majus defined by ICG angiography.

A 49 year old female patient presented a progressive lymphedema at the right leg refractory to conservative measures. In the medical history 4 years ago a laparoscopic hysterectomy and adnexectomy as well as a radical pelvic lymphadenectomy on the right side were performed because of a uterine cervical carcinoma. Despite conservative treatment the lymphedema exacerbated resulting in functional impairment and loss of quality of life. After inconspicuous follow up care and lymphoscintigraphy scan a microsurgically transplanted omentum majus flap containing lymph nodes and lymph vessles was planned. Using a laparoscopic approach the omentum majus flap was raised including the right gastroepiploic artery and vein. The flap was then anastomosed to the right femoral artery and vein. Figure 2 shows the ICG measurement after anastomosis. The well-perfused vessel arcades via the right gastroepiploic artery could be defined, whereas ICG angiography revealed the mal-perfused parts of the omentum majus which could not be determined by clinical signs. Especially in free flaps where no skin is included and peripheral bleeding on the wound edges is not common as well as residual perfusion is not sufficient for tissue survival, conventional clinical judgement by means of capillary refill or color change is not a reliable option. Discarding of too much or too less tissue is the possible consequence in these cases. Finally after discarding mal-perfused tissue parts the omentum majus was placed and spread out in the subcutaneous tissue to enable lymph vessels to grow in and establish a new lymph collector for the right lower extremity.

### Case 3

In a 55 year old female patient invasive breast cancer was diagnosed in her right breast. Neoadjuvant chemotherapy was recommended followed by modified radical mastectomy and radiotherapy. After an uneventful follow up period without tumor relapse during 1 year after mastectomy the patient presented for autologous breast reconstruction with abdominal tissue. A preoperative computed tomography angiography showed a strong lateral perforator from the inferior epigastric artery. Finally a so called DIEP flap (deep inferior epigastric artery perforator flap) was harvested based on the aforementioned lateral perforator on the left side of the abdomen. Because it was hypothesized that lateral located perforators do not constantly perfuse the flap tissue across the midline and often a possibly large flap volume is necessary especially in thin patients, ICG measurement is used to define the perfusion pattern.

**Figure 3:**
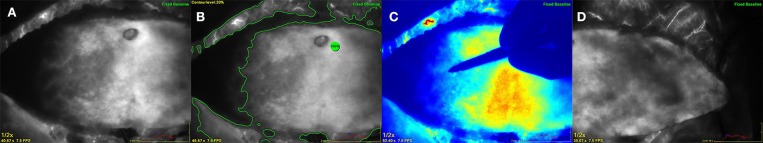
Deep inferior epigastric perforator flap after complete harvesting at the abdomen based on a single lateral located perforator. **(A)** (left) ICG angiography showing the perfusion pattern on the contralateral side of the perforator across the midline. **(B)** (middle left) Analysis by using a contour level of 20% in relation to a reference point of maximum fluorescence within the flap. **(C)** (middle right) Color mode showing the surgeons hand marking the flap borders due to the ICG perfusion pattern. **(D)** (right) ICG angiography of the ipsilateral part of the flap indicating excellent perfusion.

In this case ICG angiography showed a well perfused flap area across the midline and flap shaping was performed due to the ICG dye uptake to gain the maximum flap tissue. After anastomosis of the flap to the internal mammary artery and vein in an end-to-end fashion the repeated ICG measurement presented patent anastomoses and a well perfused DIEP flap also in the peripheral zones with no relevant change of the blood flow pattern (Figure 3).

### Case 4

**Figure 4:**
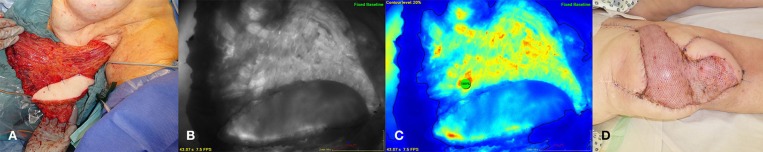
Myocutaneous latissimus dorsi flap for coverage of a defect at the right knee. **(A)** Latissimus flap after complete harvesting at the left axilla. **(B)** (middle left) ICG angiography revealing mal-perfused flap areas at the periphery. **(C)** (middle right) Color mode and contour level at 20% in relation to a reference point of maximum fluorescence within the flap. **(D)** (right) Flap after inset and anastomosis at the right knee.

An 83 year old female patient presented with a skin necrosis at the right knee and an infection of her knee joint prosthesis after multiple operations necessitating replacement of the joint prosthesis due to relapsing implant infections in the past. Because a total knee arthrodesis was not possible due to relevant shortening of the lower extremity and a high risk of osteomyelitis, wound conditioning using negative pressure wound therapy and defect reconstruction was planned to salvage the knee prosthesis and to prevent limb amputation as an ultima ratio procedure. In an interdisciplinary approach with the department of orthopedic surgery the mobile parts of the prosthesis were changed and the defect was closed with a free myocutaneous latissimus dorsi flap. The whole latissimus dorsi muscle was harvested with a large cutaneous flap island as this was necessary due to the defect size. Also in this case ICG measurement was done after harvesting and after anastomosis to the superficial femoral artery and vein. Based on the perfusion analysis the very peripheral parts of the muscle had to be discarded, whereas the cutaneous island showed normal dispersion of the dye indicating normal perfused tissue (Figure 4).

## Discussion

Due to technical advancements and the increased knowledge in the field of plastic reconstructive surgery within the last 2–3 decades, the possibilities of microsurgically free tissue transfers have increased massively. As a result, free tissue transfers on the one hand and pedicled flaps on the other hand are now so called standard procedures in a high volume center almost independent of the patient's age (2, 3). In particular, plastic reconstructive surgeons have dealt with the question of flap monitoring since the beginning of microsurgery. In the early 1980s plastic surgeons were already aware of the importance of flap monitoring, which then relied on clinical observation (8). It is generally accepted that nowadays the intraoperative analysis of flap perfusion becomes more and more important in addition to the preoperative perfusion assessment, e.g., the so-called perforator mapping (1, 9–12). Besides the perfusion assessment after flap harvesting and anastomosis, the intraoperative use of imaging techniques is of particular interest in complex constellations of the donor vessels, especially when large flaps are required that challenge the classical perforasomes. It has added a highly valuable tool for intraoperative flap shaping and designing the flaps according to their optimal perfusion zones. Only few imaging methods are actually evaluated and established in clinical routine (9–11, 13, 14). The most common methods today are the laser assisted Indocyanine Green angiography, thermography, combined laser Doppler spectrophotometry, the laser speckle contrast imaging, hyperspectral imaging or near infrared spectroscopy, respectively (15–22). Most techniques are predominantly used in the context of experimental or clinical studies and have their own advantages and disadvantages in the context of the abovementioned fields of application. The ideal monitoring instrument should primarily meet the following requirements: non-invasive and contactless, spatial resolution about the flap, accurate, and easy to use even for inexperienced personnel and providing timely information (17).

ICG angiography has been used for more than 50 years in the clinical assessment of cardiovascular function, hepatic clearance, and retinal angiography. Up to now, it is well-standardized and the laser technologies as well as the analysis options have evolved significantly. The ICG dye itself has proofed to be safe and it features for assessing tissue perfusion have enabled to develop other applications in various surgical procedures, especially in plastic surgery (10).

Denneny et al. performed an experimental study in 1983 (8) using neurovascular island flaps in rats which were transected and re-anastomosed after flap harvesting. Hereafter Fluorescein dye was injected and uptake and elimination of the dye was visualized by a fiberoptic perfusion fluorometer. They stated that this method provides reliable data about flap perfusion and predicts viability. In recent years more studies concerning tissue perfusion analysis were published (23–25). Especially in the field of immediate alloplastic breast reconstruction the perfusion of the mastectomy skin flap was evaluated using ICG angiography, because judgement of inadequate microcirculatory perfusion can be difficult and may result in skin flap or fat necrosis and reoperation. Nevertheless, for the application in free flap surgery there is still a lack of literature.

As presented in our study the advantages of this method, however, are obvious. ICG angiography offers a real-time determination of tissue vascularity and perfusion, showing a high sensitivity especially in the arterial phase of dye distribution (13). The selected representative cases prove the reproducible practicability for intraoperative perforasome definition in perforator flaps and individualized flap tailoring as well as for the judgement of muscle flaps or even the omentum majus flap.

In autologous breast reconstruction after mastectomy due to breast cancer, microsurgically transplanted tissue from the abdomen, the so-called DIEP- or muscle sparing TRAM flap, represents the gold standard today. Particularly in obese patients or in DIEP flaps where the perforator distribution and the vascular pattern within the fat layer is not predictable, clinical flap tailoring according to the zonal classification by Hartrampf or Holm might be misleading (26, 27). As shown in case 3, individual flap planning is possible by ICG angiography with a high sensitivity for mal-perfused tissue parts. Therefore, partial flap necrosis or fat necrosis can be reduced.

As we could observe in our patients, the initial phase of dye uptake within the flap tissue can be defined as safely perfused tissue. The method is independent of the investigator, reproducible as well as contactless and with a high spatial and temporal resolution. It is an external device and therefore the use is not limited to the operating room. Due to the short half-life, multiple measurements are possible at short intervals. Additionally it can be applied for postoperative flap monitoring in critical cases or if flap perfusion is not distinct by using other methods.

The application possibilities in plastic reconstructive surgery are manifold. Thus, ICG angiography can be applied not only to assess skin or muscle flaps, but also to chimeric flaps, e.g., osteomyocutaneous as well as free vascularized bone grafts such as the free medial femoral condyle bone graft or even after finger replantation (28, 29). If the technique can also contribute to judge flap autonomisation and further clarify the long term vascular changes in transplanted flaps in the long term run seems evident, but is not completely clear yet (30, 31). Further studies are required to address this topic.

However, some inherent limitations regarding ICG angiography need to be discussed also. It represents an invasive procedure in which the intravenous application of a dye is required. Throughout the years of experience with ICG the incidence of adverse effects of the dye has been infrequent and they have generally been of low severity (32, 33). Relative contraindications such as iodide allergy or severe renal failure have to be considered. In addition, the venous phase after dye uptake in the tissue is not comparable in its precision to the very sensitive arterial uptake phase. Thus, the judgement of a venous congestion is more difficult and requires more general experiences with the technique. In addition increasing background fluorescence develops in the tissue when measurements are taken repeatedly in short intervals. The latter can lead to an indistinct perfusion assessment. By using ICG angiography intraoperatively different factors could potentially influence skin perfusion. These included variables such as the use of epinephrine containing tumescent solution, administration of vasopressors, blood pressure, oxygen saturation, fraction of inspired oxygen, temperature and hematocrit as well as individual anatomical differences (34).

Despite the broad application, still no absolute or relative perfusion levels correlating with fluorescence intensity of the dye—which would allow defining parts of the tissue as well-perfused or mal-perfused, respectively—have been standardized. In our experience, a “relative perfusion level” between 20 and 30% in relation to the most fluorescent area within the relevant tissue turned out to be safely perfused tissue. Concerning the postoperative monitoring the non-continuous nature of capturing data and the large size of the device we used the application for a routine bedside monitoring postoperatively is limited (35). Nevertheless, frequent measurements are possible in principle also on the ward or in an outpatient setting.

## Conclusion

ICG angiography enables a standardized and reproducible real-time angiography of different tissues and provides a high sensitivity in detecting well-perfused and non-perfused tissue in the field of plastic reconstructive surgery. It has the potential to reduce flap related complications and to optimize and extend the possibilities of free flap surgery. Further studies in different applications fields are required to standardize application and enhance the possibilities of this method in plastic surgery.

## Ethics Statement

Written informed consent was obtained from all patients in accordance with the Declaration of Helsinki and ICG measurements were performed within a study protocol which was approved by the institutional Review Board (registration number 85_13 B).

## Author Contributions

IL: study conception. IL, RH, AA, and MS: acquisition of data. IL, RH, and MS: analysis and interpretation. IL and RH: drafting of manuscript. IL, RH, AA, and MS: critical revision.

### Conflict of Interest Statement

IL received once-only an honorary for a lecture by Novadaq but has no conflict of interest concerning this study. The remaining authors declare that the research was conducted in the absence of any commercial or financial relationships that could be construed as a potential conflict of interest.

## References

[1] Chae MP Hunter-Smith DJ Rozen WM. Comparative analysis of fluorescent angiography, computed tomographic angiography and magnetic resonance angiography for planning autologous breast reconstruction. Gland Surg. (2015) 4:164–78. 10.3978/j.issn.2227-684X.2015.03.06 26005648 PMC4409669

[2] Ludolph I Lehnhardt M Arkudas A Kneser U Pierer G Harder Y. [Plastic reconstructive microsurgery in the elderly patient - consensus statement of the German Speaking Working Group for Microsurgery of the Peripheral Nerves and Vessels]. Handchir Mikrochir Plast Chir. (2018) 50:118–25. 10.1055/s-0043-115730 29045998

[3] Fried FW Beier JP Bohr C Iro H Horch RE Arkudas A. Free latissimus dorsi myocutaneous flap in a 6-month-old child for reconstruction of a temporal fossa defect after teratoma resection. Ann Plast Surg. (2019) 82:62–3. 10.1097/SAP.0000000000001629 30285989

[4] Steiner D Horch RE Eyupoglu I Buchfelder M Arkudas A Schmitz M. Reconstruction of composite defects of the scalp and neurocranium-a treatment algorithm from local flaps to combined AV loop free flap reconstruction. World J Surg Oncol. (2018) 16:217. 10.1186/s12957-018-1517-0 30404625 PMC6223072

[5] Mathes DW Neligan PC. Current techniques in preoperative imaging for abdomen-based perforator flap microsurgical breast reconstruction. J Reconstruct Microsurg. (2010) 26:3–10. 10.1055/s-0029-1244806 20024888

[6] Lohman RF Ozturk CN Ozturk C Jayaprakash V Djohan R. An analysis of current techniques used for intraoperative flap evaluation. Ann Plast Surg. (2015) 75:679–85. 10.1097/SAP.0000000000000235 25003438

[7] Liu DZ Mathes DW Zenn MR Neligan PC. The application of indocyanine green fluorescence angiography in plastic surgery. J Reconstruct Microsurg. (2011) 27:355–64. 10.1055/s-0031-1281515 21717392

[8] Denneny JC III Weisman RA Silverman DG. Monitoring free flap perfusion by serial fluorometry. Otolaryngol Head Neck Surg. (1983) 91:372–6. 10.1177/019459988309100405 6415582

[9] Bellamy JL Mundinger GS Flores JM Wimmers EG Yalanis GC Rodriguez ED. Do adjunctive flap-monitoring technologies impact clinical decision making? an analysis of microsurgeon preferences and behavior by body region. Plast Reconstruct Surg. (2015) 135:883–92. 10.1097/PRS.0000000000001064 25719704

[10] Burnier P Niddam J Bosc R Hersant B Meningaud JP. Indocyanine green applications in plastic surgery: a review of the literature. J Plast Reconstruct Aesthet Surg. (2017) 70:814–27. 10.1016/j.bjps.2017.01.020 28292569

[11] Smit JM Negenborn VL Jansen SM Jaspers MEH de Vries R Heymans MW. Intraoperative evaluation of perfusion in free flap surgery: a systematic review and meta-analysis. Microsurgery. (2018) 38:804–18. 10.1002/micr.30320 29577423

[12] Saint-Cyr M Wong C Schaverien M Mojallal A Rohrich RJ. The perforasome theory: vascular anatomy and clinical implications. Plast Reconstruct Surg. (2009 124:1529–44. 10.1097/PRS.0b013e3181b98a6c 20009839

[13] Ludolph I Arkudas A Schmitz M Boos AM Taeger CD Rother U Cracking the perfusion code?: Laser-assisted Indocyanine Green angiography and combined laser Doppler spectrophotometry for intraoperative evaluation of tissue perfusion in autologous breast reconstruction with DIEP or ms-TRAM flaps. J Plast Reconstruct Aesthet Surg. (2016) 69:1382–8. 10.1016/j.bjps.2016.07.014 27522453

[14] Hauck T Horch RE Schmitz M Arkudas A. Secondary breast reconstruction after mastectomy using the DIEP flap. Surg Oncol. (2018) 27:513. 10.1016/j.suronc.2018.06.006 30217311

[15] Beier JP Horch RE Arkudas A Dragu A Schmitz M Kneser U. Decision-making in DIEP and ms-TRAM flaps: the potential role for a combined laser Doppler spectrophotometry system. J Plast Reconstruct Aesthet Surg. (2013) 66:73–9. 10.1016/j.bjps.2012.08.040 23017936

[16] Rothenberger J Amr A Schaller HE Rahmanian-Schwarz A. Evaluation of a non-invasive monitoring method for free flap breast reconstruction using laser doppler flowmetrie and tissue spectrophotometry. Microsurgery. (2013) 33:350–7. 10.1002/micr.22096 23436443

[17] Holmer A Marotz J Wahl P Dau M Kammerer PW. Hyperspectral imaging in perfusion and wound diagnostics - methods and algorithms for the determination of tissue parameters. Biomed Tech Biomed Eng. (2018) 63:547–56. 10.1515/bmt-2017-0155 30028724

[18] Holm C Mayr M Hofter E Dornseifer U Ninkovic M. Assessment of the patency of microvascular anastomoses using microscope-integrated near-infrared angiography: a preliminary study. Microsurgery. (2009) 29:509–14. 10.1002/micr.20645 19306390

[19] Hardwicke JT Osmani O Skillman JM. Detection of perforators using smartphone thermal imaging. Plast Reconstruct Surg. (2016) 137:39–41. 10.1097/PRS.0000000000001849 26710006

[20] Draijer M Hondebrink E van Leeuwen T Steenbergen W. Review of laser speckle contrast techniques for visualizing tissue perfusion. Lasers Med Sci. (2009) 24:639–51. 10.1007/s10103-008-0626-3 19050826 PMC2701498

[21] Briers D Duncan DD Hirst E Kirkpatrick SJ Larsson M Steenbergen W. Laser speckle contrast imaging: theoretical and practical limitations. J Biomed Opt. (2013) 18:066018. 10.1117/1.JBO.18.6.066018 23807512

[22] Rother U Gerken ALH Karampinis I Klumpp M Regus S Meyer A. Dosing of indocyanine green for intraoperative laser fluorescence angiography in kidney transplantation. Microcirculation. (2017) 24:1–7. 10.1111/micc.12392 28787770

[23] Casey WJ III Connolly KA Nanda A Rebecca AM Perdikis G Smith AA. Indocyanine green laser angiography improves deep inferior epigastric perforator flap outcomes following abdominal suction lipectomy. Plast Reconstruct Surg. (2015) 135:491e–7e. 10.1097/PRS.0000000000000964 25719713

[24] Swanson E. Comparison of limited and full dissection abdominoplasty using laser fluorescence imaging to evaluate perfusion of the abdominal skin. Plast Reconstruct Surg. (2015) 136:31e–43e. 10.1097/PRS.0000000000001376 26111330

[25] Preidl RH Schlittenbauer T Weber M Neukam FW Wehrhan F. Assessment of free microvascular flap perfusion by intraoperative fluorescence angiography in craniomaxillofacial surgery. J Cranio-Max-Facial Surg. (2015) 43:643–8. 10.1016/j.jcms.2015.03.013 25913628

[26] Holm C Mayr M Hofter E Ninkovic M. Perfusion zones of the DIEP flap revisited: a clinical study. Plast Reconstruct Surg. (2006) 117:37–43. 10.1097/01.prs.0000185867.84172.c0 16404245

[27] Hartrampf CR Scheflan M Black PW Breast reconstruction with a transverse abdominal island flap. Plast Reconstruct Surg. (1982) 69:216–25. 10.1097/00006534-198202000-00007 6459602

[28] Gould DJ Mehrara BJ Neligan P Cheng MH Patel KM. Lymph node transplantation for the treatment of lymphedema. J Surg Oncol. (2018) 118:736–42. 10.1002/jso.25180 30129675

[29] Valente SA Al-Hilli Z Radford DM Yanda C Tu C Grobmyer SR. Near infrared fluorescent lymph node mapping with indocyanine green in breast cancer patients: a prospective trial. J Am Coll Surg. (2018) 228:672–678. 10.1016/j.jamcollsurg.2018.12.001 30582975

[30] Granzow J Li AI Caton A Boyd JB. Free flap survival following failure of the vascular pedicle. Ann Plast Surg. (2015) 75:44–8. 10.1097/SAP.0000000000000136 25643188

[31] Yoon AP Jones NF. Critical time for neovascularization/angiogenesis to allow free flap survival after delayed postoperative anastomotic compromise without surgical intervention: a review of the literature. Microsurgery. (2016) 36:604–12. 10.1002/micr.30082 27375230

[32] Newman MI Samson MC. The application of laser-assisted indocyanine green fluorescent dye angiography in microsurgical breast reconstruction. J Reconstruct Microsurg. (2009) 25:21–6. 10.1055/s-0028-1090617 18925547

[33] Hope-Ross M Yannuzzi LA Gragoudas ES Guyer DR Slakter JS Sorenson JA. Adverse reactions due to indocyanine green. Ophthalmology. (1994) 101:529–33. 10.1016/S0161-6420(94)31303-0 8127574

[34] Munabi NC Olorunnipa OB Goltsman D Rohde CH Ascherman JA. The ability of intra-operative perfusion mapping with laser-assisted indocyanine green angiography to predict mastectomy flap necrosis in breast reconstruction: a prospective trial. J Plast Reconstruct Aesthet Surg. (2014) 67:449–55. 10.1016/j.bjps.2013.12.040 24507962

[35] Karinja SJ Lee BT. Advances in flap monitoring and impact of enhanced recovery protocols. J Surg Onco. (2018) 118:758–67. 10.1002/jso.25179 30132901

